# English proficiency and global competence among undergraduates in Sino-Foreign Cooperation Programmes: the mediating role of intercultural sensitivity and moderating role of English learning motivation

**DOI:** 10.3389/fpsyg.2026.1809017

**Published:** 2026-07-22

**Authors:** Huizhen Wang, Sijia Cheng, Michael T. C. Hwang, Ruilan Cao

**Affiliations:** 1 Jimei University, Xiamen, China; 2 Anhui University of Technology, Ma'anshan, China; 3 Huaqiao University, Quanzhou, China

**Keywords:** English learning motivation, English proficiency, global competence, intercultural sensitivity, SFCPs

## Abstract

English proficiency is positively associate with students' global competence. However, the mechanism underlying the relationship remains unclear. To address this gap, drawing upon social cognitive theory, intercultural sensitivity theory, and self-determination theory, this study examined the mediating role of intercultural sensitivity and the moderating role of English learning motivation in the relationship between English proficiency and global competence among undergraduates enrolled in Sino-Foreign Cooperation Programmes (SFCPs). A cross-sectional survey was conducted among 420 undergraduates from specific SFCP institutions, and the data were analyzed using Partial Least Squares Structural Equation Modeling (PLS-SEM). Results revealed that English proficiency was positively associated with global competence (β = 0.427, *p* < 0.001), with intercultural sensitivity partially mediating this relationship (β = 0.169, *p* < 0.001). Additionally, English learning motivation was found to moderate the relationship between English proficiency and global competence (β = 0.233, *p* < 0.001) as well as the relationship between English proficiency and intercultural sensitivity (β = 0.303, *p* < 0.001). These findings suggest that students' global competence is not only directly linked to English proficiency but also indirectly associated through intercultural sensitivity and moderated by motivational factors. Given the cross-sectional design and the limited sampling frame, this study offers preliminary empirical insights rather than broad generalizations. The results imply that integrating language development, intercultural training, and motivational support within SFCPs may be beneficial, though further longitudinal research is required to establish causality and test the generalisability of these findings across diverse educational contexts.

## 1 Introduction

In recent years, the cultivation of global competence has become a key focus in higher education policy worldwide, especially in the context of internationalized programmes (Abelha et al., 2020; Majewska, 2023; Tours and Lynch, 2023), particularly within the context of Sino-Foreign Cooperation Programmes (SFCPs) at China Universities (Han, 2023; Zhuang et al., 2024). As a distinctive model of international education, Sino-Foreign Cooperative Education (SFCE) plays a crucial role in advancing the internationalization of Chinese higher education and has become an integral part of China's education system (Ying and Quan, 2023). However, despite the increasing emphasis on global competence in education, many undergraduates in SFCPs demonstrate relatively limited levels of global competence (Zha and Wu, 2021), which impedes their ability to thrive in diverse cultural settings and hinders their overall academic and professional development (Zhou, 2022). This issue is particularly pronounced in regions or institutions where students have limited opportunities for immersive intercultural experiences, making it more essential to understand how internal factors such as motivation and sensitivity compensate for these contextual limitations (Shadiev et al., 2024).

While Chinese SFCPs have provided strategic platforms for integrating global learning objectives into local curricula, research suggests that they tend to prioritize structural compliance and curriculum transplantation, which may sometimes limit opportunities for deeper intercultural engagement (Hu, 2014; Zhu, 2022). By contrast, international models such as Erasmus+ in Europe (Grebe, 2023), the AAC&U global learning framework in the U.S. (Bozeman et al., 2023), Australia's New Colombo Plan (Australian Government, 2022), and Japan's G30 initiative highlight transformative practices (Rees, 2024), such as student mobility, multicultural teamwork, and immersive exchange, that have proven effective in cultivating global competence. Taken together, these international experiences indicate that the cultivation of global competence requires not only supportive institutional design but also sustained attention to individual-level attributes such as language proficiency (Risner, 2021), intercultural sensitivity (Lee Olson and Kroeger, 2001), and motivation (Semaan and Yamazaki, 2015). Thus, to contextualize the Chinese case globally, this study investigates how these personal factors contribute to competence development among SFCP undergraduates.

### 1.1 The relationship between English proficiency and global competence

A growing body of research highlights English proficiency as a crucial determinant of undergraduates' global competence, particularly in the context of Sino-Foreign Cooperation Programmes (SFCPs), where English serves both as a medium of instruction and as a symbolic marker of international readiness (Han, 2023; Zhuang et al., 2024). Numerous studies confirm that English proficiency is positively associated with global competence (Garcia-Perez et al., 2014; Huang, 2021; Cao and Meng, 2020). Similar mechanisms linking linguistic proficiency and global competence have been observed in other English-as-a-foreign-language (EFL) contexts. For instance, studies across Japan (Sakamoto and Roger, 2023), South Korea (Lee, 2024), and ASEAN countries (Sarwari and Wahab, 2018) indicate that English proficiency enhances global competence and global citizenship. Beyond these comparable EFL settings, research in European context (Zhang et al., 2025) and American context (Garcia-Perez et al., 2014) further demonstrates that language proficiency can nurture global citizenship. For instance, Cao and Meng (2020) surveyed 555 Chinese students and found that English achievement was positively correlated with global competence, with motivation acting as a moderator, while Guo et al. (2024) demonstrated in a large-scale cross-national study that both foreign language acquisition and use predicted global competence. These findings suggest that English proficiency not only facilitates communication but also provides access to international knowledge and participation in global discourse (Sattar, 2017; Arkoudis et al., 2009). Yet, scholars also caution that linguistic ability alone does not guarantee intercultural understanding or a global mindset (Lou et al., 2022).

However, building on these insights, recent work has called for disaggregating English proficiency into its component skills when examining its relationship with global competence. Listening competence is associated with intercultural adaptability and comprehension of diverse accents (Schaefer and Darcy, 2021; Liu and Zhang, 2023; Irizarry Quintero et al., 2024), reading enables access to global academic and cultural resources that are conducive to critical thinking (Reimers, 2009; Hoff, 2019; Short et al., 2023; Al-Jarf, 2022), while writing and translating cultivate intercultural mediation and expression across languages (Liddicoat, 2016; Arslan, 2020). Accordingly, the present study operationalises English proficiency using CET-4 scores across three components—listening, reading, and writing/translating—to assess their distinct contributions to global competence among SFCP undergraduates, thereby laying the foundation for the hypotheses advanced in this study (Hou and Huang, 2024; Zhang, 2024; Huang, 2023).

### 1.2 Intercultural sensitivity as a mediator between English proficiency and global competence

Intercultural sensitivity is regarded as a key factor linking language proficiency to global competence (Katitaş et al., 2024). Numerous studies report a positive relationship between English proficiency and intercultural sensitivity (Alijanian et al., 2019; Wang and Huang, 2013). For instance, Alijanian et al. (2019) confirmed a significant correlation among Iranian EFL learners, and Wu (2013) found similar results with Chinese undergraduates, suggesting that higher proficiency promotes openness and empathy toward diverse cultures. However, inconsistent results exist: Wu (2016) reported no significant association among Taiwanese learners, highlighting the influence of context and measurement.

Meanwhile, intercultural sensitivity has been repeatedly associated with global competence. Ernawati et al. (2022) found significant correlations in a study of health professionals; Mostafaei Alaei and Nosrati (2018) reported similar findings among EFL teachers; and Sarwari and Abdul Wahab (2017) demonstrated strong links with intercultural communication competence among international postgraduates. These results suggest that intercultural sensitivity is linked to intercultural communication and global perspectives. Furthermore, its mediating role has been supported in related domains, such as between cultural intelligence and multicultural attitudes (Akcaoglu and Kayiş, 2021; Basman and Bayram, 2024), multicultural education attitudes (Katitaş et al., 2024), and implicit intercultural identification (He et al., 2023).

### 1.3 English learning motivation as a moderator between English proficiency and global competence

Motivation is another crucial factor shaping language learning and global readiness. According to Dörnyei's (2009) L2 Motivational Self System, both the “ideal L2 self” and the “ought-to self” sustain engagement, thereby enhancing proficiency. Empirical evidence confirms strong positive links between motivation and English proficiency: Iwaniec (2022) found that Spanish adolescents with integrative motivation achieved superior multiskill performance; Harrison and Rodriguez (2023) reported a strong correlation (*r* = 0.68, *p* < 0.001) among U.S. high school students; and Luo (2021) showed that intrinsically motivated Chinese learners sustained proficiency gains, whereas extrinsically motivated peers stagnated. Yet some findings diverge, such as Ilma's (2018) study in Indonesia, which revealed no significant relationship, likely due to heterogeneous motivational types.

Motivation is correlated with global competence. Kim (2021) identified positive links across four motivational components among Chinese freshmen; Semaan and Yamazaki (2015) reported similar findings in U.S. critical language classrooms; and Zhou et al. (2023) highlighted that in SFCPs, highly motivated learners benefit more from internationalized curricula and faculty. Beyond direct effects, motivation has been shown to act as a moderator: Cao and Meng (2020) reported that motivation moderated the relationship between personality traits and global competence among Chinese students; Shafi et al. (2020) and Zhou et al. (2023) found similar patterns in cross-cultural educational contexts. These findings underscore the role of motivation in strengthening the relationship between proficiency and global competence.

### 1.4 English learning motivation as a moderator between English proficiency and intercultural sensitivity

Recent studies indicate that English learning motivation is positively associated with intercultural sensitivity. Huyen-Thanh (2022) found that motivated EFL undergraduates in Vietnam demonstrated higher intercultural sensitivity, while Liu and Zhang (2023) reported similar correlations among international students learning Chinese, suggesting that motivation fosters openness and empathy in intercultural contexts. Theoretically, Dörnyei's (2009) L2 Motivational Self System explains that learners with a strong Ideal L2 Self are more likely to engage in intercultural communication, while Bennett's (1986) DMIS highlights motivation as an enabling factor for progression from ethnocentrism to ethnorelativism, further supported by later work (Lamy et al., 2013; Tajeddin and Alemi, 2021).

Although empirical evidence supports a direct motivation–sensitivity link, little research has tested motivation's moderating role between proficiency and intercultural sensitivity. Scholars argue that language proficiency alone may not ensure intercultural competence without motivation to engage with diversity (Oberste-Berghaus, 2024). Thus, students with similar proficiency levels may vary considerably in intercultural awareness depending on motivational dispositions. In SFCPs, where students must navigate both linguistic and cultural boundaries, motivation may strengthen the relationship between English proficiency and intercultural sensitivity.

### 1.5 Key constructs

In this study, global competence is conceptualized following the OECD (2018) as the capacity to examine local, global, and intercultural issues, to understand and appreciate the perspectives of others, to engage in effective and respectful interactions across cultures, and to take responsible action for collective well-being. English proficiency is defined as multiskill competence across listening, speaking, reading, writing, and translation in the Chinese context (Gustanti and Ayu, 2021). Intercultural sensitivity is understood as the affective capacity to recognize, respect, and adapt to cultural differences, fostering openness and empathy in intercultural encounters (Sitron and Lock, 2021). Finally, English learning motivation refers to the psychological drive that influences students' willingness, effort, and persistence in acquiring English as a second language (Ramzan et al., 2023).

### 1.6 The present study

Based on the aforementioned literature and the existing research gap, and with a specific focus on undergraduates in Sino-foreign cooperation programmes, the present study aims to investigate the relationships between individual English proficiency, intercultural sensitivity, English learning motivation, and global competence. Specifically, we seek to address three research questions (RQs): (1) Can English proficiency contribute to the global competence of undergraduates in Sino-foreign cooperation programme? (2) Can intercultural sensitivity mediate the relationship between English proficiency and global competence? (3) Can English learning motivation moderate the relationship between English proficiency and global competence? (4) Can English learning motivation moderate the relationship between English proficiency and intercultural sensitivity? Accordingly, the hypotheses were proposed as follows:

**Hypothesis 1**. English proficiency is positively correlated with students' global competence.

**Hypothesis 2**. Intercultural sensitivity can mediate the relationship between English proficiency and global competence.

**Hypothesis 3**. English learning motivation can moderate the relationship between English proficiency and global competence.

**Hypothesis 4**. English learning motivation can moderate the relationship between English proficiency and intercultural sensitivity.

**Figure 1:**
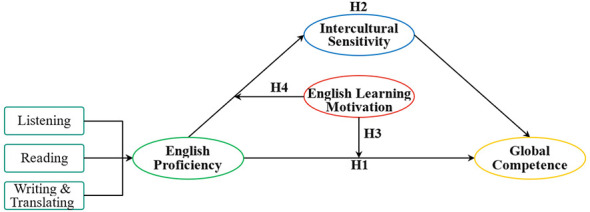
Hypothesized model.

Building on the aforementioned research questions and hypotheses, this study proposes a hypothesized model (Figure 1), explaining the relationships between English proficiency, intercultural sensitivity, English learning motivation, and global competence. The model is grounded in Social Cognitive Theory, Intercultural Sensitivity Theory, and Self-Determination Theory. Social Cognitive Theory (Bandura, 2003) posits triadic reciprocity among person, behavior, and environment; in this study, English proficiency (EP) is a personal capability shaped by learning contexts, while intercultural sensitivity (IS) and global competence (GC) are behavioral outcomes that grow through observation and authentic cross-cultural practice. Intercultural Sensitivity Theory (Bennett, 1986) conceptualizes IS as a progression from ethnocentrism to ethnorelativism, positioning IS as a mediator that translates EP into GC by enabling recognition, adaptation, and effective interaction across cultures. Self-Determination Theory (Deci et al., 2017; Ryan and Deci, 2020) emphasizes autonomous motivation as the volitional engine of sustained engagement; accordingly, English learning motivation (ELM) moderates the EP → IS and EP → GC links by amplifying the extent to which linguistic resources are activated in intercultural settings. Together, these lenses specify a coherent mechanism comprising a direct path (EP → GC), an indirect path (EP → IS → GC), and moderated effects whereby higher ELM strengthens both links (Figure 1).

## 2 Methods

### 2.1 Participants and procedures

The intended target population consisted of undergraduates from Sino-Foreign Cooperation Programmes in China who had taken the College English Test Band 4 (CET-4). Due to accessibility, feasibility, and resource constraints (Andrade, 2020), the operational sampling frame was restricted to programmes in Fujian Province.

A multi-stage sampling strategy was employed to ensure a representative sample of undergraduates enrolled in Sino-Foreign Cooperation Programmes in Fujian Province. At Stage 1, the province was stratified into four geographic strata (North, South, East, and West) to account for potential variation across regions. The South stratum was then purposively selected based on accessibility and feasibility. At Stage 2, within the selected stratum, academic programmes were treated as cluster units. Two programmes—International Accounting and Business Administration—were chosen according to the following criteria: accessibility to the researcher, student participation in the CET-4 test, and voluntary consent to participate. At Stage 3, from the eligible 3rd-year and 4th-year students in each programme (approximately 900 undergraduates who had completed at least 2 years of English instruction and sat for CET-4), individuals were drawn using stratified random sampling by academic year, followed by simple random sampling within each stratum. In total, 420 students were selected (210 per programme, equally divided between Year 3 and Year 4). This design combined stratification (Stage 1), cluster sampling (Stage 2), and stratified-simple random sampling (Stage 3) to enhance sample diversity and reduce selection bias within the participating programmes. However, because the South stratum and participating programmes were selected partly on the basis of accessibility and feasibility, the sample should not be considered statistically representative of all SFCP undergraduates in China.

Among the 420 respondents, 214 were female (51.0%) and 206 were male (49.0%), showing a nearly balanced gender distribution. Most students (78.3%) were aged 21–22, while 11.7% were 23 or older, and fewer were below 20 years (10.0%). The respondents were evenly drawn from two programmes—International Accounting (*n* = 210) and Business Administration (*n* = 210)—and equally split between Year 3 and Year 4. In terms of language background, the majority spoke Mandarin at home (82.1%), followed by Cantonese (13.6%) and English (4.3%). Most had studied English for more than 10 years (72.1%), while 25.0% had 6–9 years of learning experience, and only 2.9% reported 4–6 years. Regarding overseas experience, 80.7% reported having lived abroad. In this study, overseas experience referred to actual residence in a foreign country or region for educational, exchange, family, or related purposes and did not include online international experiences or virtual exchange activities. Most students reported overseas residence of 6–12 months (43.1%), while smaller proportions reported ≤ 6 months (26.4%) or ≥1 year (11.2%). The main reasons for learning English were academic purposes (64.5%) and professional development (62.6%), with smaller proportions citing travel (27.1%), personal interest (21.9%), or cultural understanding (13.1%).

Permission to collect data was granted by the two programmes on 31 August 2024. Ethical approval was obtained from the institutional ethics committee in UCSI University (Reference code: IEC-2024-FOSSLA-0142) on 18 October 2024. All voluntary respondents were fully informed the study's purpose and procedures, who were assured that all data were encrypted and stored securely on the Sojump platform (Wenjuanxing), accessible only to authorized researchers. The study followed a three-phase design conducted between October 2024 and March 2025. First, three experts validated the questionnaire content. Second, a pilot study was conducted in one programme to refine the instruments. Finally, the full survey was administered online through Sojump to randomly selected undergraduates from another two programmes.

### 2.2 Measures

***English proficiency*.** The College English Test Band 4 (CET-4) score was used to assess the undergraduates' English proficiency because it is a compulsory high-stakes, large scale, and nationwide English proficiency test to measure English proficiency of non-English majors (Li, 2021; Yan and Huizhong, 2006). The test allocates 35% to listening, 35% to reading, and 30% to writing and translating, with a total score of 710 (Li, 2021), in which the scoring criteria for the CET-4 were established following the latest reforms in 2016.

***Intercultural sensitivity*.** Intercultural sensitivity was measured using the Intercultural Sensitivity Scale (ISS) developed by Chen and Starosta (2000). The scale includes 24 items across five dimensions: interaction engagement (seven items, e.g., “I enjoy interacting with people from different cultures.”), respect for cultural differences (six items, e.g., “I respect the ways people from different cultures behave.”), interaction confidence (five items, “I am pretty sure of myself in interacting with people from different cultures.”), interaction enjoyment (three items, e.g., “I don't get upset easily when interacting with people from different cultures.”), and interaction attentiveness (three items, e.g., “I am sensitive to my culturally-distinct counterpart's subtle meanings during our interaction.”). The scale demonstrated good reliability (α = 0.88). Items were rated on a five-point Likert scale, with higher scores indicating stronger intercultural sensitivity.

***English learning motivation*.** The Intrinsic/Extrinsic Motivation Scale of English Learning (I/EMSEL) developed by Wang (2008) was used to elicit data on the undergraduates' English learning motivation. The scale includes 21 items across two dimensions: intrinsic motivation (12 items, e.g., “I will persist when facing difficulties in learning English.”) and extrinsic motivation (nine items, e.g., “In order to know the recent development in my major, I study English diligently”). Reliability was acceptable (α = 0.80). Responses were rated on a five-point Likert scale, with higher scores reflecting greater motivation. The I/EMSEL is grounded in Self-Determination Theory (Deci and Ryan, 1985) and has been widely applied in language learning research.

***Global competence*.** Global competence was measured using the Global Competence Scale (GCS) developed by Liu et al. (2020). The scale consists of 33 items across three dimensions: knowledge and understanding (nine items, α = 0.879, e.g., “Apart from my own country, I know about the history and geography of at least one other country.”), skills (13 items, α = 0.867, e.g., “I can easily use MS Office, PDF Reader, and other common international softwares.”), and attitudes and values (11 items, α = 0.907, e.g., “When communicating with foreigners, I try to respect their cultures and values.”). The overall reliability was high (α = 0.920). Responses were rated on a five-point Likert scale (1 = strongly disagree, 5 = strongly agree). The GCS has been validated in Chinese higher education and aligns with OECD's global competence framework (Jiang et al., 2023).

Taken together, all variables in this study except for English proficiency were assessed using self-report questionnaires. While these instruments have demonstrated acceptable levels of reliability and validity, the exclusive reliance on self-reported data may introduce response bias (Caputo, 2017), which constitutes a methodological limitation and should be considered in future research.

### 2.3 Data analysis

The data were analyzed using Statistical Package for the Social Sciences (SPSS) Version 27.0 (IBM Corp., Armonk, NY, United States) and SmartPLS Version 4.0 (SmartPLS GmbH, Oststeinbek, Germany). This study was conducted in six steps to ensure methodological rigor and validity. First, preliminary analyses were performed, including data cleaning, missing data treatment, common method bias checks, normality assessment, and multicollinearity diagnostics. Second, descriptive statistics using SPSS summarized demographic characteristics. Third, the measurement model was assessed in SmartPLS by examining indicator reliability, internal consistency, convergent validity, and discriminant validity. Fourth, the structural model was evaluated through collinearity diagnostics, path coefficient significance (via bootstrapping with 5,000 resamples), explanatory power (*R*^2^), effect size (*f* ^2^), predictive relevance (*Q*^2^), and PLSpredict for out-of-sample prediction. Fifth, mediation analysis tested the indirect effect of intercultural sensitivity between English proficiency and global competence. Finally, moderation analysis assessed whether English learning motivation moderated the relationships of English proficiency with global competence and intercultural sensitivity using interaction terms and simple slope analysis. This integrated approach ensured robust testing of the hypothesized relationships.

## 3 Results

### 3.1 Common method bias test results

To assess the potential presence of common method bias (CMB), this study employed two tests: the Harman's single-factor test and the full collinearity test. In the Harman's test, all items were subjected to exploratory factor analysis. The first unrotated factor accounted for 23.929% of the total variance, well below the 50% threshold (Podsakoff et al., 2003), indicating that CMB is not a serious concern. Additionally, the full collinearity test revealed that all variance inflation factors (VIFs) for endogenous constructs were below three (Hair et al., 2011), with the highest VIF being 1.967, further confirming that these tests reduce concerns regarding common method bias.

### 3.2 Measurement model evaluation results

The measurement model was evaluated following the four standard criteria for reflective measurement in PLS-SEM: indicator reliability, internal consistency reliability, convergent validity, and discriminant validity (Hair et al., 2022), to ensure construct validity before structural model analysis.

First, the indicator reliability was assessed through outer loadings of the measurement items. As detailed in Table 1, the item-level outer loadings ranged from 0.716 to 0.916, all surpassing the recommended threshold of 0.708, indicating satisfactory item reliability and that no indicators required deletion or modification (Hair et al., 2022). Second, internal consistency was evaluated using both Cronbach's alpha (α) and Composite Reliability (CR). As shown in Table 1, all α values ranged from 0.732 to 0.948, exceeding the 0.70 benchmark recommended by DeVellis and Thorpe (2021), indicating acceptable internal consistency. Similarly, all CR values were above 0.70, with the lowest CR value being 0.857, further confirming strong internal consistency reliability.

**Table 1:** Internal consistency reliability and convergent validity indicators.

Construct	Item	Outer loading	S.E	*t*	*P*	Cronbach's alpha	CR (*ρ_*c*_*)	AVE
First order								
English proficiency	LI	0.865	0.016	54.976	0.000	0.813	0.889	0.728
	RE	0.866	0.017	50.729	0.000			
	WT	0.827	0.024	34.969	0.000			
Interaction engagement	ENG1	0.802	0.020	41.102	0.000	0.906	0.925	0.639
	ENG2	0.814	0.020	40.112	0.000			
	ENG3	0.794	0.026	31.026	0.000			
	ENG4	0.760	0.030	25.339	0.000			
	ENG5	0.783	0.027	29.319	0.000			
	ENG6	0.811	0.022	36.502	0.000			
	ENG7	0.829	0.020	40.677	0.000			
Respect of cultural difference	RCD1	0.851	0.014	58.839	0.000	0.909	0.930	0.687
	RCD2	0.860	0.016	52.793	0.000			
	RCD3	0.821	0.021	39.481	0.000			
	RCD4	0.811	0.023	35.325	0.000			
	RCD5	0.828	0.021	39.491	0.000			
	RCD6	0.802	0.024	33.701	0.000			
Interaction confidence	CO1	0.819	0.018	45.526	0.000	0.882	0.914	0.679
	CO2	0.827	0.020	41.288	0.000			
	CO3	0.793	0.023	34.670	0.000			
	CO4	0.837	0.019	43.331	0.000			
	CO5	0.844	0.019	43.486	0.000			
Interaction enjoyment	ENJ1	0.843	0.016	53.252	0.000	0.817	0.891	0.732
	ENJ2	0.869	0.014	63.376	0.000			
	ENJ3	0.854	0.017	49.712	0.000			
Interaction attractiveness	AT1	0.856	0.015	56.855	0.000	0.792	0.878	0.706
	AT2	0.829	0.019	44.765	0.000			
	AT3	0.836	0.019	43.736	0.000			
Knowledge and understanding	KU1	0.785	0.023	34.460	0.000	0.935	0.945	0.659
	KU2	0.805	0.020	39.318	0.000			
	KU3	0.736	0.029	25.351	0.000			
	KU4	0.804	0.022	36.714	0.000			
	KU5	0.822	0.020	41.491	0.000			
	KU6	0.823	0.020	40.708	0.000			
	KU7	0.858	0.017	49.691	0.000			
	KU8	0.836	0.019	44.135	0.000			
	KU9	0.829	0.019	42.662	0.000			
Skills	SK1	0.732	0.029	25.484	0.000	0.948	0.954	0.617
	SK10	0.752	0.026	29.120	0.000			
	SK11	0.916	0.012	76.837	0.000			
	SK12	0.792	0.024	33.387	0.000			
	SK13	0.784	0.024	32.140	0.000			
	SK2	0.799	0.021	37.986	0.000			
	SK3	0.818	0.020	41.186	0.000			
	SK4	0.758	0.026	29.107	0.000			
	SK5	0.779	0.023	34.356	0.000			
	SK6	0.755	0.027	27.837	0.000			
	SK7	0.721	0.030	24.030	0.000			
	SK8	0.791	0.025	31.955	0.000			
	SK9	0.799	0.025	32.412	0.000			
Attitudes and values	AV1	0.751	0.026	29.429	0.000	0.923	0.934	0.564
	AV10	0.783	0.022	35.403	0.000			
	AV11	0.730	0.028	25.716	0.000			
	AV2	0.780	0.022	35.387	0.000			
	AV3	0.758	0.027	28.239	0.000			
	AV4	0.762	0.027	28.642	0.000			
	AV5	0.765	0.026	29.060	0.000			
	AV6	0.720	0.029	24.457	0.000			
	AV7	0.729	0.027	27.082	0.000			
	AV8	0.734	0.027	26.726	0.000			
	AV9	0.745	0.026	28.947	0.000			
Intrinsic motivation	IM1	0.772	0.027	28.518	0.000	0.928	0.938	0.559
	IM10	0.755	0.027	28.365	0.000			
	IM11	0.736	0.033	22.096	0.000			
	IM12	0.805	0.022	36.637	0.000			
	IM2	0.789	0.023	34.788	0.000			
	IM3	0.736	0.029	25.299	0.000			
	IM4	0.730	0.028	25.649	0.000			
	IM5	0.725	0.031	23.193	0.000			
	IM6	0.726	0.031	23.404	0.000			
	IM7	0.716	0.033	21.957	0.000			
	IM8	0.717	0.034	20.873	0.000			
	IM9	0.761	0.027	28.149	0.000			
Extrinsic motivation	EM1	0.819	0.021	38.721	0.000	0.934	0.945	0.655
	EM2	0.814	0.022	37.217	0.000			
	EM3	0.838	0.018	45.733	0.000			
	EM4	0.803	0.025	32.309	0.000			
	EM5	0.801	0.025	32.684	0.000			
	EM6	0.834	0.021	40.571	0.000			
	EM7	0.830	0.022	38.510	0.000			
	EM8	0.738	0.031	24.034	0.000			
	EM9	0.801	0.023	34.230	0.000			
Second order								
Intercultural sensitivity	ENG	0.769	0.025	30.320	0.000	0.828	0.879	0.593
	RCD	0.810	0.022	36.512	0.000			
	CO	0.763	0.026	29.048	0.000			
	ENJ	0.759	0.024	30.998	0.000			
	AT	0.747	0.028	26.711	0.000			
Global competence	KU	0.788	0.023	34.443	0.000	0.750	0.857	0.667
	SK	0.840	0.017	50.271	0.000			
	AV	0.821	0.018	45.862	0.000			
English learning motivation	IM	0.887	0.011	83.649	0.000	0.732	0.882	0.788
	EM	0.889	0.010	86.295	0.000			

CR, composite reliability; AVE, average variance extracted.

Third, convergent validity was assessed using the Average Variance Extracted (AVE). All AVE values exceeded the 0.50 threshold, with the minimum AVE being 0.559, indicating satisfactory convergent validity (Hair et al., 2022). Fourth, discriminant validity was evaluated using cross-loadings, the Heterotrait–Monotrait Ratio (HTMT), and the Fornell–Larcker criterion. Cross-loading analysis confirmed that each indicator loaded higher on its intended construct than on other constructs. For HTMT, all values ranged between 0.087 and 0.718 for higher-order constructs, well below the conservative cut-off of 0.85 (Hair et al., 2022), as presented in Table 2.

**Table 2:** HTMT ratios for discriminant validity assessment.

Construct	Intercultural sensitivity	Global competence	English learning motivation	English proficiency
Intercultural sensitivity				
Global competence	0.683			
English learning motivation	0.087	0.119		
English proficiency	0.674	0.718	0.069	

### 3.3 Structural model evaluation results

The structural model was evaluated, following the confirmation of measurement model reliability and validity, to assess the explanatory power and predictive capability of the proposed relationships. As shown in Table 3, the model demonstrated acceptable explanatory power, with *R*^2^ values of 0.442 for intercultural sensitivity and 0.456 for global competence. According to Cohen (2013), *R*^2^ values of 0.02, 0.13, and 0.26 can be interpreted as weak, moderate, and substantial, respectively; therefore, the current model demonstrates substantial explanatory power for both endogenous constructs.

**Table 3:** Assessment of the structural model.

Estimated model	*R* ^2^	*f* ^2^	*Q* ^2^
Intercultural sensitivity	0.442	EP → IS = 0.458	0.256
Global competence	0.456	EP → GC = 0.225	0.296
		IS → GC = 0.033	

EP, English proficiency; IS, intercultural sensitivity; GC, global competence.

Regarding effect sizes, English proficiency showed a large effect on intercultural sensitivity (*f* ^2^ = 0.458), and a medium effect on global competence (*f* ^2^ = 0.225). Intercultural sensitivity exhibited a small effect on global competence (*f* ^2^ = 0.033). These effect sizes are consistent with Cohen's (2013) benchmarks, where *f* ^2^ values of 0.02, 0.15, and 0.35 represent small, medium, and large effects, respectively. Thus, these results support the substantive significance of the hypothesized relationships.

Furthermore, the predictive relevance of the model was evaluated using Stone-Geisser's *Q*^2^ values, yielding *Q*^2^ = 0.256 for intercultural sensitivity and *Q*^2^ = 0.296 for global competence. Both values exceed the threshold of zero, indicating satisfactory predictive relevance for the endogenous constructs (Hair et al., 2014).

### 3.4 Correlation analyses

To answer RQ1, path coefficients were examined to assess the direct relationships between English proficiency and global competence. The results revealed that English proficiency had a positive and significant direct association with global competence (β = 0.427, *t* =8.286, *p* = 0.000, *f* ^2^ = 0.225). Following Cohen's (2013) guidelines (*f*^2^: small = 0.02, medium = 0.15, large = 0.35), the observed effect (*f*^2^ = 0.225) can be interpreted as medium to approaching large, suggesting that English proficiency makes a meaningful contribution to their perceived global competence.

In addition, English proficiency demonstrated significant positive associations with intercultural sensitivity (β = 0.510, *t* = 12.416, *p* = 0.000, *f* ^2^ = 0.458), indicating a large effect. This finding highlights the strong positive association between English proficiency and students' intercultural sensitivity. Moreover, intercultural sensitivity also showed a significant positive association with global competence (β = 0.179, *t* = 2.881, *p* = 0.004, *f* ^2^ = 0.033), which constitutes a small effect. While statistically significant, this indicates that intercultural sensitivity plays a more modest role in its association with global competence compared to English proficiency. These preliminary associations laid the foundation for subsequent mediation and moderation analyses. Table 4 presents the detailed correlation results among the study variables.

**Table 4:** Correlations of the study variables.

Path	β	S.E	*t*	*p*	95%CI		*f* ^2^	VIF	Supported
					Lower	Upper			
EP → GC	0.427	0.051	8.286	0.000	0.327	0.530	0.225	1.483	Yes
IS → GC	0.179	0.062	2.881	0.004	0.051	0.295	0.033	1.792	Yes
ELM → GC	0.030	0.043	0.685	0.493	−0.055	0.112	0.002	1.009	No
EP → IS	0.510	0.041	12.416	0.000	0.430	0.589	0.458	1.017	Yes
ELM → IS	0.003	0.046	0.068	0.946	−0.089	0.087	0.000	1.009	No

β, standardized path coefficient; S.E., standard error; *t, t*-statistic; *p, p*-value; CI, 95% confidence interval; VIF, variance inflation factor; EP, English proficiency; IS, intercultural sensitivity; GC, global competence; ELM, English learning motivation.

### 3.5 Testing for mediation effect

This section addressed RQ2 by examining the mediating role of intercultural sensitivity in the relationship between English proficiency and global competence. Following the standard two-step procedure for mediation analysis, the direct and indirect effects were assessed. Table 5 presents the results of the mediation analysis.

**Table 5:** Mediation effect results.

Correlation type	Path	β	*t*	*p*	RQ	Decision	Mediation type
Direct effects	EP → GC	0.427	8.286	0.000	RQ2	Supported	Partial mediation
	LI → GC	0.159	3.006	0.003			
	RE → GC	0.176	3.058	0.002			
	WT → GC	0.167	3.412	0.001			
	EP → IS	0.510	12.416	0.000			
	IS → GC	0.179	2.881	0.004			
Indirect effects	EP → IS → GC	0.169	4.750	0.000			
	LI → IS → GC	0.086	3.971	0.000			
	RE → IS → GC	0.072	3.132	0.002			
	WT → IS → GC	0.052	2.634	0.008			

β, standardized path coefficient; *t, t*-statistic; *p, p*-value; EP, English proficiency; GC, global competence; IS, intercultural sensitivity; LI, listening; RE, reading; WT, writing and translating.

First, as for the direct effect, English proficiency has a statistically significant and positive direct association with global competence (β = 0.427, *t* = 8.286, *p* = 0.000), This finding suggests that individuals with higher English proficiency tend to achieve a higher global competence. Additionally, English proficiency is positively related to intercultural sensitivity (β = 0.510, *t* = 12.416, *p* = 0.000). These results indicate that English proficiency is positively linked to intercultural sensitivity. Furthermore, intercultural sensitivity is significantly related global competence (β = 0.179, *t* = 2.881, *p* = 0.004). When looking at the three dimensions of English proficiency, all have significant and positive relationship with global competence. Specifically, listening proficiency is associated with global competence (β = 0.159, *t* = 3.006, *p* = 0.003), reading proficiency also showed a significant association (β = 0.176, *t* = 3.058, *p* = 0.002), and writing and translating proficiency had a positive and significant association (β = 0.167, *t* = 3.412, *p* = 0.001). These findings collectively highlight that different dimensions of English proficiency are correlated with students' global competence.

As for the indirect effects (mediating effect), the mediation analysis results show that intercultural sensitivity partially mediates the relationship between English proficiency and global competence. The overall indirect effect of English proficiency on global competence through intercultural sensitivity is statistically significant (β = 0.169, *t* = 4.750, *p* = 0.000). Further analysis of the dimensions revealed that listening (β = 0.086, *t* = 3.971, *p* = 0.000), reading (β = 0.072, *t* = 3.132, *p* = 0.002), and writing and translating (β = 0.052, *t* = 2.634, *p* = 0.008) all showed significant indirect effects on global competence via intercultural sensitivity.

Partial mediation was determined following the criteria outlined by Hair et al. (2022), whereby both the direct (β = 0.427, *t* = 8.286, *p* = 0.000) and indirect (β = 0.169, *t* = 4.750, *p* = 0.000) effects remained statistically significant. These findings answer RQ2 and revealed the mediating role of intercultural sensitivity in enhancing the positive relationship between English proficiency (including its sub-dimensions of listening, reading, and writing and translating) on global competence.

### 3.6 Testing for moderation effect

This section addresses RQ3 and RQ4 by examining whether English learning motivation moderates the relationship between English proficiency and both global competence and intercultural sensitivity. Regarding the direct effects, as shown in Table 6, English proficiency showed a strong positive association with both global competence (β = 0.427, *p* < 0.001) and intercultural sensitivity (β = 0.510, *p* < 0.001). Intercultural sensitivity also positively predicted global competence (β = 0.179, *p* = 0.004). However, the direct paths from English learning motivation to either intercultural sensitivity (β = 0.003, *p* = 0.946) or global competence (β = 0.030, *p* = 0.493) were non-significant, suggesting that motivation alone does not directly enhance these outcomes.

**Table 6:** Moderating effect results.

RQ	Path	β	S.E.	*t*	*p*	95%CI		Decision
						Lower	Upper	
	EP → GC	0.427	0.051	8.286	0.000	0.327	0.530	
	EP → IS	0.510	0.041	12.416	0.000	0.430	0.589	
	IS → GC	0.179	0.062	2.881	0.004	0.051	0.295	
	ELM → IS	0.003	0.046	0.068	0.946	−0.089	0.087	
	ELM → GC	0.030	0.043	0.685	0.493	−0.055	0.112	
RQ3	ELM × EP → GC	0.233	0.040	5.817	0.000	0.162	0.318	Supported
RQ4	ELM × EP → IS	0.303	0.037	8.168	0.000	0.236	0.380	Supported

β, standardized path coefficient; *t, t*-statistic; *p, p*-value; CI, 95% confidence interval; EP, English proficiency; GC, global competence; IS, intercultural sensitivity; ELM, English learning motivation.

As for the moderation effects, the interaction terms demonstrated significant moderating effects. The interaction between English learning motivation and English proficiency on global competence was positive and significant (β = 0.233, *p* < 0.001). Similarly, the interaction between motivation and proficiency on intercultural sensitivity was also positive and significant (β = 0.303, *p* < 0.001). These results indicate that higher levels of learning motivation strengthen the positive associations of English proficiency with both intercultural sensitivity and global competence.

**Figure 2:**
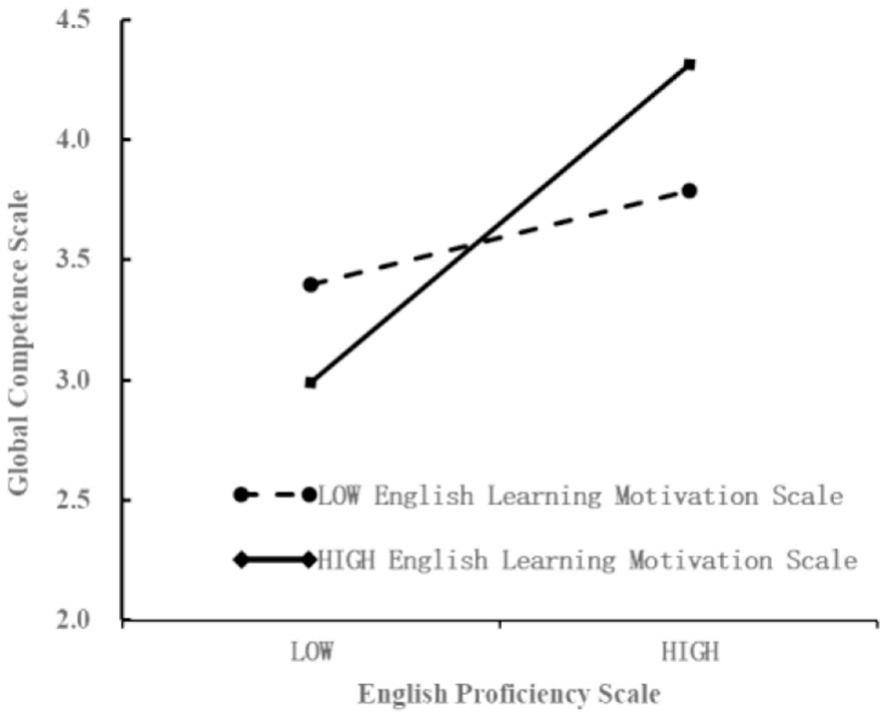
Slope plot of ELM in the relationship between EP and GC.

**Figure 3:**
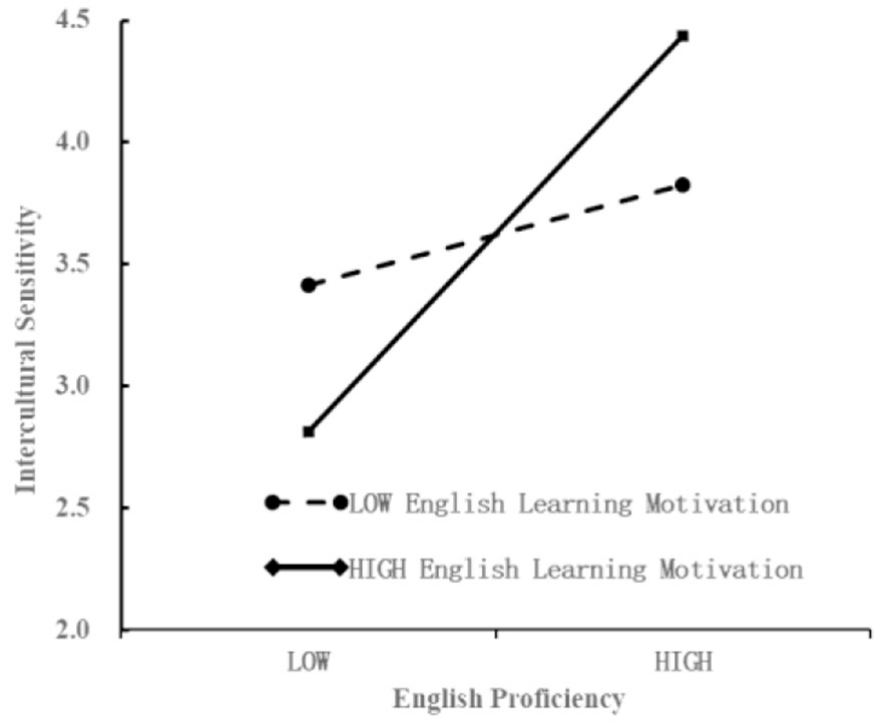
Slope plot of ELM in the relationship between EP and IS.

To visually illustrate the moderation effect, Figures 2, 3 present the simple slope analyses. Figure 2 shows the moderating role of English learning motivation (ELM) in the relationship between English proficiency (EP) and global competence (GC). The slope for students with high motivation (+1 SD) was much steeper, indicating that proficiency strongly enhances global competence when motivation is high. In contrast, the slope for low motivation (−1 SD) was relatively flat, suggesting that proficiency contributes only weakly to global competence under low motivational conditions. Figure 3 illustrates the moderating effect of ELM on the relationship between EP and intercultural sensitivity (IS). Similarly, the slope for high motivation was significantly steeper, highlighting that motivated students translate their proficiency into higher intercultural sensitivity more effectively, whereas low motivation dampens this relationship.

To summarize, these findings underscore the pivotal role of motivation in shaping how English proficiency contributes to intercultural sensitivity and global competence. When students possess high levels of learning motivation, their language proficiency more effectively translates into stronger intercultural skills and broader global competence. Conversely, low motivation substantially weakens these links, suggesting that proficiency alone is insufficient without motivational drive. Overall, the results highlight that fostering motivation is essential for maximizing the benefits of English proficiency in developing both intercultural sensitivity and global competence.

## 4 Discussion

### 4.1 English proficiency and global competence

The findings demonstrate a positive and significant association between English proficiency and global competence, consistent with some prior empirical research (Garcia-Perez et al., 2014; Cao and Meng, 2020). This suggests that English language ability plays a fundamental role in equipping students to navigate the demands of globalization. Beyond a skills-based account, this pattern can also be interpreted as a form of the cultural and social capitals transfer (Wei et al., 2011), where English proficiency functions as both symbolic and practical capital that affords entry to global discourses, epistemic resources, and cosmopolitan networks, which are essential to intercultural interactions (Pham and Tran, 2015). Also, these findings are aligned with Social Cognitive Theory (Bandura, 2003), which posits that personal capabilities (such as language skills) interact with environmental influences (such as cross-cultural academic exposure) to shape behavior and higher-order competencies like global competence. In the specific context of Sino-Foreign Cooperation Programmes, where students are immersed in bilingual and bicultural learning environments, English proficiency thus operates both as a communicative capability and as an access right to global learning opportunities, helping explain the medium-to-large effect sizes observed.

To further unpack this relationship, English proficiency was deconstructed into three key dimensions: listening, reading, and writing and translating. Listening proficiency showed a strong association with global competence, indicating that students who can effectively comprehend spoken English are more likely to develop intercultural decoding skills and empathy for diverse communicative styles (Schaefer and Darcy, 2021). These findings resonate with Liu and Zhang (2023), who showed that listening skills are positively associated with intercultural sensitivity and global mindset among Chinese undergraduates. Reading proficiency also showed a significant association with global competence, underscoring reading as a gateway to global knowledge and intercultural understanding. Students with strong reading ability access authentic materials, global news, policy documents, and international research, that that are associated with broader worldviews and greater critical literacy (Short et al., 2023). Finally, writing and translating were also significantly associated with global competence. Writing enables intercultural argumentation, while translation fosters metalinguistic awareness and perspective-taking (Liddicoat, 2016; OECD, 2018).

Importantly, this multidimensional view of proficiency offers an enriched understanding compared to much of the international literature, which often relies on composite test scores (e.g., TOEFL/IELTS) as single indicators (Coney and Isbell, 2026). In China's Sino-Foreign Cooperation Programmes, however, students frequently shuttle between Chinese and English academic discourses, making translation skills particularly salient.

### 4.2 Mediating role of intercultural sensitivity

The mediation analysis results revealed that intercultural sensitivity partially mediates the relationship between English proficiency and global competence. This sheds light on a previously unexplored aspect within the literature. This finding contributes to the literature by clarifying the mechanism through which English proficiency translates into broader global competencies. While earlier studies mainly examined the direct EP–GC relationship (Garcia-Perez et al., 2014; Cao and Meng, 2020), the current study shows that intercultural sensitivity plays an indispensable psychological role.

This aligns with both Social Cognitive Theory (Bandura, 2003) and Intercultural Sensitivity Theory (Bennett, 1986), which emphasize that language skills provide the resources for interaction, while intercultural sensitivity transforms these interactions into meaningful global engagement. Students with higher proficiency are more likely to engage with international curricula, foreign faculty, and multicultural peers, thereby developing intercultural awareness (Wu, 2016).

In China, this finding carries particular significance. Although English-medium instruction has become widespread, Chinese students may still lack genuine intercultural encounters beyond the classroom (Xiuwen and Razali, 2020). By contrast, Western students often acquire intercultural sensitivity through study-abroad programmes or everyday multicultural interactions (Tarchi et al., 2019). The partial mediation effect (17% of the total EP–GC effect) observed here suggests that without intentional interventions—such as experiential learning, global simulation projects, and reflection-based intercultural training—the transformative potential of English proficiency in China could remain underutilized. Thus, language instruction alone cannot guarantee global competence; it must be integrated with intercultural pedagogy (Lou et al., 2022; Liddicoat, 2016). Sino-Foreign Cooperation Programmes should therefore embed experiential and reflective practices, enabling students to move from linguistic access to intercultural agency (Trede et al., 2013).

### 4.3 Moderating role of motivation in EP and GC

The results confirm that English learning motivation significantly moderates the relationship between English proficiency and global competence. Students with higher motivation demonstrated a stronger ability to leverage their language skills for global readiness, while low-motivation students showed a weaker link. The slope analysis visualized this moderation, highlighting that proficiency without motivation may not result in genuine intercultural engagement (Oberste-Berghaus, 2024).

Additionally, this finding resonates with Self-Determination Theory (Deci et al., 2017), which stresses that motivated learners are more likely to persist, adapt, and engage meaningfully in intercultural contexts. When motivation is more autonomously regulated, learners show greater persistence, curiosity, and reflective processing, thereby amplifying the transfer of linguistic resources into intercultural outcomes. This also aligns with the L2 Motivational Self System, in which a salient “Ideal L2 Self” energizes students to seek out intercultural interactions where sensitivity and global competence are cultivated (Dörnyei, 2009). From an investment perspective, motivated learners are more willing to risk face, time, and effort to enter communities of practice where both intercultural sensitivity and global competence can develop.

Empirical studies (Cao and Meng, 2020; Zhou et al., 2023; Kim, 2021) similarly emphasize that motivation amplifies the role of proficiency in fostering global competence. Notably, the non-significant direct paths from motivation to competence observed in this study are theoretically plausible: motivation is not a direct competence predictor *per se* but rather an activating condition or volitional catalyst that determines whether proficiency is effectively mobilized in intercultural settings. In other words, proficiency provides the “capacity” for intercultural engagement, while motivation supplies the “willingness” to activate that capacity; the synergy between the two is essential, as motivation without proficiency may result in unfulfilled intentions, and proficiency without motivation may remain an underutilized resource (Oberste-Berghaus, 2024). From a comparative perspective, Chinese undergraduates often approach English learning with strong extrinsic motivations (e.g., exams, job opportunities), while Western contexts highlight intrinsic motivations such as self-expression and international mobility. Although the present study did not find a significant direct effect of overall motivation on intercultural sensitivity or global competence, the significant moderating effects suggest that motivation quality may matter more than its quantity. Given that the motivation scale used in this study allows for the distinction between intrinsic and extrinsic orientations (Wang, 2008), future research could further examine whether intrinsic motivation, rather than extrinsic motivation, serves as a more potent moderating factor in the proficiency-competence relationship. The current findings suggest that fostering intrinsic elements, curiosity, enjoyment, global citizenship, may be crucial for maximizing the return on China's heavy investment in English education.

### 4.4 Moderating role of motivation in EP and IS

The findings also indicate that English learning motivation (ELM) significantly strengthens the pathway from English proficiency (EP) to intercultural sensitivity (IS). Theoretically, this pattern is consistent with Self-Determination Theory and the L2 Motivational Self System: motivation does not create IS as a competence; rather, it operates as a volitional catalyst that converts latent linguistic resources into approach-oriented intercultural behaviors where IS can develop (Deci et al., 2017; Dörnyei, 2009). In other words, EP provides capacity, while ELM governs the activation of that capacity in socially demanding, ambiguous, or face-threatening situations that are formative for IS.

Consistent with the findings of previous studies (Huyen-Thanh, 2022; Liu and Zhang, 2023; Kim, 2021), the present findings demonstrate that English proficiency alone may not be sufficient for cultivating intercultural sensitivity. While English proficiency provides the necessary linguistic resources for cross-cultural communication, a psychological factor like English learning motivation determines the extent to which learners will actively engage in diverse cultural perspectives and apply their language skills in authentic intercultural contexts (Oberste-Berghaus, 2024). In this regard, English learning motivation serves as a psychological driving force that amplifies the relationship between English proficiency and intercultural sensitivity, functioning as an activating condition that enables learners to translate their linguistic capacity into genuine intercultural engagement.

Moreover, the findings extend the findings of past studies (Alijanian et al., 2019; Wu, 2013) by clarifying the conditional nature of the intercultural relationship. The moderation effect observed in this study emphasizes the complex interplay between cognitive resources (language skills) and affective factors (motivation) in fostering global competence. This is particularly relevant in the context of Sino-Foreign Cooperation Programmes, where students often experience dual demands of language acquisition and intercultural adaptation (Han, 2023). In this regard, students with high motivation are more likely to actively participate in English-mediated courses, seek intercultural encounters, and overcome communication anxiety, thereby enhancing their intercultural sensitivity (Tajeddin and Alemi, 2021).

### 4.5 Theoretical implications

This study advances theory in several ways. First, the robust positive association between English proficiency and global competence among Sino-foreign undergraduates substantiates the Social Cognitive perspective that personal capabilities, such as multiskill language proficiency, transact with academic contexts to cultivate higher-order competencies (Bandura, 2003). By disaggregating English proficiency into listening, reading, and writing and translating, the study refines language–competence accounts that often rely on composite indices, showing that distinct skills make meaningful, differentiated contributions to global competence in bilingual and bicultural programmes (Schaefer and Darcy, 2021; Short et al., 2023).

Second, the partial mediation of intercultural sensitivity clarifies the psychological pathway through which English proficiency translates into global competence. Rather than language skill operating as a direct pipeline to global readiness, intercultural sensitivity functions as a socio-affective hinge that transforms linguistic access into intercultural understanding and engagement. This mechanism helps reconcile mixed findings in prior work by specifying how proficiency becomes consequential for global competence (Wu, 2016).

Third, the significant moderating roles of English learning motivation on both the English proficiency–global competence and English proficiency–intercultural sensitivity relationships integrate motivation-centric perspectives with language and intercultural theories. Motivation amplifies the yield of proficiency. Students high in English learning motivation convert similar levels of proficiency into markedly greater intercultural sensitivity and global competence. Conceptually, the findings support a model in which English proficiency is positively associated with global competence both directly and indirectly through intercultural sensitivity, while English learning motivation strengthens the relationships between English proficiency and global competence as well as between English proficiency and intercultural sensitivity (Deci et al., 2017; Dörnyei, 2009).

Together, these results culminate in a theoretically grounded model for Sino-Foreign Cooperation Programmes: English proficiency is directly associated with global competence; English proficiency is also indirectly associated with global competence through intercultural sensitivity; and English learning motivation strengthens both the direct and indirect associations. The model advances nuance beyond the notion that “more English leads to more global competence,” highlighting which skills matter, through what mechanisms, and under what motivational conditions.

### 4.6 Practical implications

The findings also carry practical significance. In curriculum design, programmes should embed intercultural pedagogy alongside language instruction, for example through reflection journals, critical incident analysis, global simulation projects, and collaborative online international learning activities, so that gains in proficiency are reliably associated with intercultural sensitivity and global competence (Trede et al., 2013; Lou et al., 2022). English proficiency should be taught and assessed as multiskill, with tasks that differentially target listening across accents, reading authentic global texts, and writing or translation for intercultural mediation (Liddicoat, 2016).

In instructional practice, motivation-sensitive design is essential. Autonomy-supportive tasks, clear value framing, and scaffolded mastery experiences can raise learning motivation, particularly for students whose motivation is primarily exam-driven. Structured intercultural contact, such as co-taught sessions with foreign faculty, peer mentoring with international students, or cross-cultural case-based projects, should also be prioritized (Zhou et al., 2023).

In terms of assessment, institutions should complement CET-4 with performance-based evaluations of global competence and intercultural sensitivity, such as role-plays, multilingual briefs, or reflective rubrics. Monitoring differential gains by motivational profile may allow targeted support for students at risk of low transfer from proficiency to competence.

At the institutional and policy level, it is necessary to expand experiential opportunities through short-term mobility, virtual internships, or international capstones, especially in contexts where physical study abroad is limited. Faculty development in intercultural pedagogy should also be incentivised, and programme evaluation frameworks should align with global competence outcomes rather than only test scores.

### 4.7 Limitations and future research

Despite its contributions, the study has several limitations. First, the model focused on four variables—English proficiency, intercultural sensitivity, motivation, and global competence. Several demographic and contextual characteristics, including gender, year of study, programme, years of English learning, home language, overseas experience, socioeconomic background, international exposure, academic self-efficacy, peer networks, and teacher competence, were not included in the present model. Although preliminary group-difference analyses were conducted for programme type and years of English learning, these variables were not incorporated as controls within the structural model. Therefore, some observed relationships may have been influenced by unmeasured contextual factors. Future studies should incorporate these characteristics as control variables or additional predictors to further examine the robustness of the proposed relationships.

Second, the study employed a cross-sectional design, limiting causal inference. Longitudinal research tracking students across academic years and into early careers would provide more robust insights into developmental processes. Moreover, except for English proficiency, all measures relied on self-reports, which may introduce response bias and limit objectivity. Future studies should incorporate multi-source data, including behavioral tasks, teacher ratings, and peer assessments, to triangulate findings (Caputo, 2017). Third, the sample was drawn from two programmes within one province and included a purposively selected geographic stratum. Therefore, the findings should be interpreted with caution and cannot be assumed to be statistically representative of all SFCP undergraduates in China. Future studies should employ probability-based sampling across multiple provinces, institutions, and disciplines to improve generalisability. Fourth, the study did not account for the role of programme-level intercultural affordances such as exposure to international faculty or participation in virtual exchange. Future studies could adopt multilevel designs to partition individual- and programme-level influences. Additionally, the significant moderating and mediating roles identified suggest the need to explicitly model moderated mediation or mediated moderation in future research.

## 5 Conclusion

Drawing upon Social Cognitive Theory, Intercultural Sensitivity Theory, and Self-Determination Theory, we proposed and examined a conceptual framework regarding the collective influence of English proficiency, intercultural sensitivity, and English learning motivation on global competence among undergraduates in Sino-Foreign programmes. We concluded that: (1) English proficiency positively and significantly influence both intercultural sensitivity and global competence; (2) intercultural sensitivity positively influence global competence and partially mediates the relationship between English proficiency and global competence; and (3) English learning motivation positively moderates the relationship between English proficiency and both global competence and intercultural sensitivity, such that the positive association is stronger among more motivated individuals. These findings suggest adopting an integrative approach that combines language instruction with intercultural training and motivation-enhancing strategies.

## Data Availability

The raw data supporting the conclusions of this article will be made available by the authors, without undue reservation.
